# Clinical and electrodiagnostic features of nerve conduction study-classified peripheral neuropathy in symptomatic patients with antineutrophil cytoplasmic antibody-associated vasculitis: a single-centre cohort study

**DOI:** 10.3389/fimmu.2026.1807774

**Published:** 2026-07-30

**Authors:** Jang Woo Ha, Oh Chan Kwon, Yong-Beom Park, Sang-Won Lee

**Affiliations:** 1 Division of Rheumatology, Department of Internal Medicine, Yongin Severance Hospital, Yonsei University College of Medicine, Yongin, Gyeonggi-do, Republic of Korea; 2 Division of Rheumatology, Department of Internal Medicine, Gangnam Severance Hospital, Yonsei University College of Medicine, Seoul, Republic of Korea; 3 Division of Rheumatology, Department of Internal Medicine, Yonsei University College of Medicine, Seoul, Republic of Korea; 4 Institute for Immunology and Immunological Diseases, Yonsei University College of Medicine, Seoul, Republic of Korea

**Keywords:** antineutrophil cytoplasmic antibody, biopsy, nerve conduction studies, peripheral neuropathy, vasculitis

## Abstract

**Background:**

This study investigated the clinical and electrodiagnostic features of vasculitic peripheral neuropathy (VPN) in symptomatic patients with antineutrophil cytoplasmic antibody-associated vasculitis (AAV) who underwent nerve conduction studies (NCS) within 3 months after AAV diagnosis.

**Methods:**

Among 323 patients with AAV, 97 presenting with clinical symptoms suggestive of VPN and with the availability of results of NCS performed within 3 months after AAV diagnosis were included in this study. VPN was classified based on the case definition and guidelines proposed by the Brighton Collaboration Vasculitic Peripheral Neuropathy Working Group in 2017. Of the 97 patients, 12 underwent nerve biopsy.

**Results:**

At AAV diagnosis, the median age of the 97 patients was 63.0 years (57% women), consisting of 48 patients with microscopic polyangiitis (MPA), 20 with granulomatosis with polyangiitis (GPA), and 29 with eosinophilic granulomatosis with polyangiitis (EGPA). Among symptomatic AAV patients who underwent early NCS, 55 patients (56.7%) were classified as having electrodiagnostically supported VPN. The most common type of VPN was a mixed type (83.6%), and the most frequently affected nerve was the peroneal nerve (70.9%). The concordance rate between NCS and nerve biopsy findings was 75% based on histological evidence of vasculitis.

**Conclusion:**

In this selected cohort of symptomatic AAV patients who underwent NCS within 3 months after diagnosis, more than half were classified as having electrodiagnostically supported VPN. NCS may provide adjunctive objective information for characterizing peripheral nerve involvement in clinically suspected VPN.

## 1 Introduction

Antineutrophil cytoplasmic antibody (ANCA)-associated vasculitis (AAV) is a group of small vessel vasculitis clinically characterised by a link between circulating ANCA in the peripheral blood and histologically fibrinoid necrotising vasculitis (1, 2). The types of vessels affected by AAV vary from capillaries alongside adjacent arterioles and venules to medium-sized arteries (1). AAV is generally categorised into three subtypes according to its clinical features: microscopic polyangiitis (MPA), granulomatosis with polyangiitis (GPA), and eosinophilic granulomatosis with polyangiitis (EGPA) (3–6). Meanwhile, based on the type of ANCA, it may be divided into three subtypes: myeloperoxidase (MPO)-ANCA vasculitis, proteinase 3 (PR3)-ANCA vasculitis, and ANCA-negative vasculitis (7). Theoretically, AAV can involve and damage almost any organ, and the Birmingham vasculitis activity score version 3 (BVAS), five-factor score (FFS), and vasculitis damage index (VDI) forms were developed to assess AAV activity, prognosis, and extent of damage, and include nine items based on systemic manifestations according to each unique compartment (8–10).

Among these various systemic manifestations of AAV, peripheral neuropathy (PN) caused by vasculitis (vasculitic peripheral neuropathy, VPN) has been reported in up to 50% of AAV patients (11, 12). In real clinical practice, VPN is classified using objective clinical, electrodiagnostic, and/or histological evidence according to the Brighton Collaboration case definition, which was developed in the context of prior diagnostic frameworks, including the Peripheral Nerve Society guideline for vasculitic neuropathy (13, 14). Nerve biopsy provides the most direct histological evidence of vasculitic involvement of peripheral nerves, but it is invasive and therefore cannot be routinely recommended for all AAV patients exhibiting clinical symptoms suggestive of VPN, such as multifocal or asymmetric pain, tingling sensation, numbness, and/or weakness in upper or lower extremities. Therefore, non-invasive nerve conduction studies (NCS) are performed more frequently and widely in real clinical practice (15–17). Clinical assessment, NCS, and appropriate imaging studies also help distinguish peripheral neuropathy from radiculopathy or other mimicking conditions. Nerve biopsy may be considered selectively when histological confirmation of vasculitic neuropathy is clinically necessary and feasible (18, 19).

Given that VPN is a severe life/organ-threatening cases of AAV that requires aggressive induction therapy with rituximab and/or cyclophosphamide (20, 21), it should be classified earlier and more accurately in AAV patients. Furthermore, for the early classification of VPN, information on the frequency and clinical characteristics of VPN should be investigated and a consensus regarding the diagnostic methods for VPN classification should be established in AAV patients. However, data on early electrodiagnostic findings in symptomatic AAV patients with suspected VPN remain limited. Therefore, in the present study, we included AAV patients who presented with clinical symptoms suggestive of VPN and had NCS results available within 3 months after AAV diagnosis. We aimed to describe the proportion and clinical characteristics of electrodiagnostically supported VPN in this selected symptomatic cohort and to evaluate the potential adjunctive role of NCS in characterizing peripheral nerve involvement.

## 2 Methods

### 2.1 Patients

**Figure 1:**
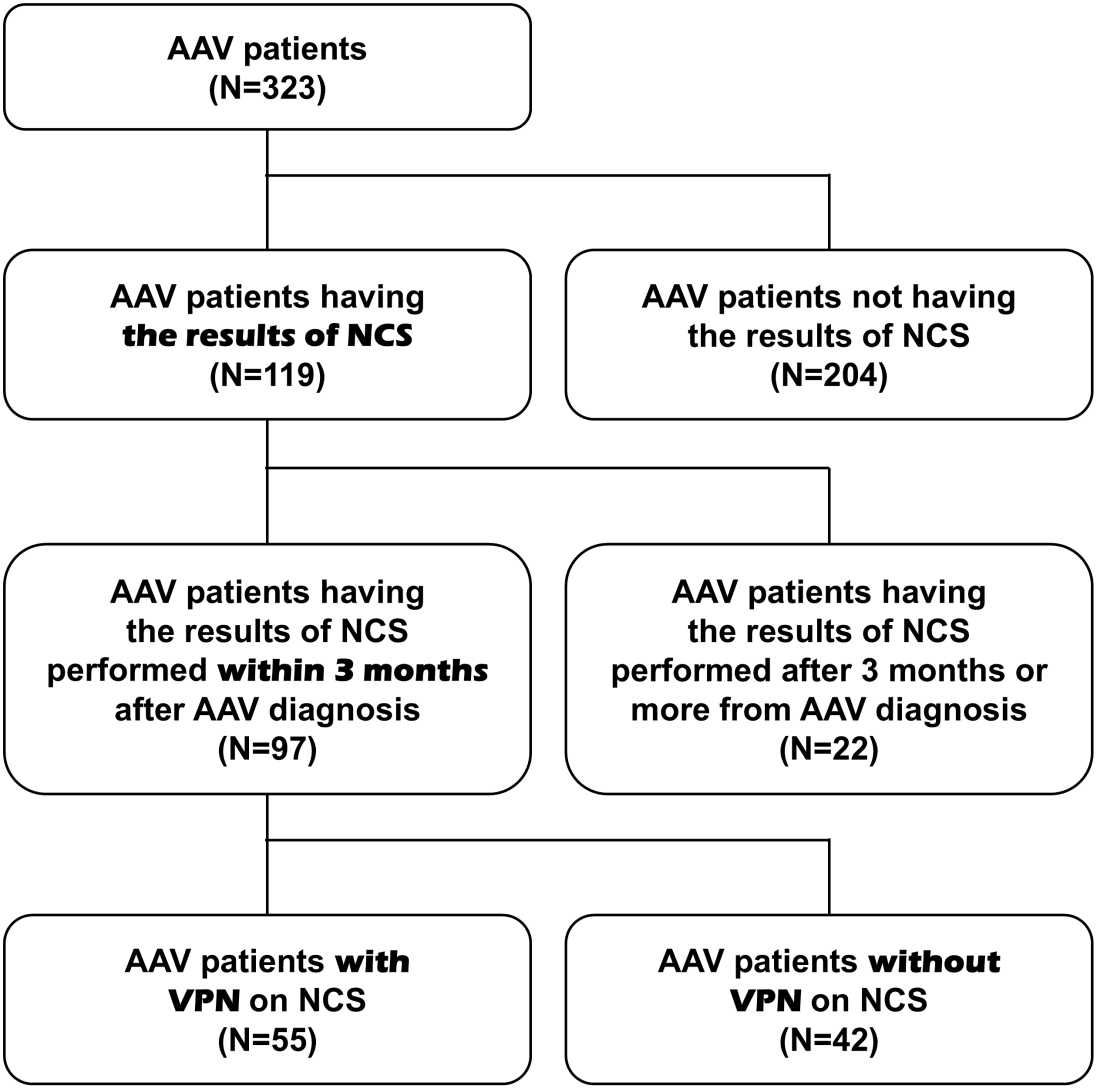
Inclusion and exclusion of patients with AAV. AAV, antineutrophil cytoplasmic antibody-associated vasculitis; NCS, nerve conduction studies; VPN, vasculitic peripheral neuropathy.

We retrospectively reviewed the medical records of 323 patients enrolled in the Severance Hospital ANCA-associated Vasculitides cohort, a single-centre observational cohort of Korean patients with AAV. The inclusion criteria were as follows: 1) patients who were first classified as having AAV at this hospital and confirmed by rheumatologists; 2) patients who were classified based on the revised 2012 Chapel Hill Consensus Conference Nomenclature of Vasculitides (1); 3) patients who met the algorithm for AAV and polyarteritis nodosa proposed by the European Medicine Agency in 2007 (the EMA algorithm), and the classification criteria for MPA, GPA, and EGPA proposed by a joint group of the American College of Rheumatology and the European Alliance of Associations for Rheumatology in 2022 (the ACR/EULAR criteria) (2–6); 4) patients who had well-written medical records that included clinical, laboratory, radiological, and histological information for classifying AAV according to both the EMA algorithm and ACR/EULAR criteria (2–5); 5) patients who had available ANCA results for perinuclear (P)-ANCA/cytoplasmic (C)-ANCA as well as MPO-ANCA/PR3-ANCA within 3 months before and after AAV diagnosis (22); 6) patients who had been follow-up for 3 months or more after AAV diagnosis; 7) patients who had no serious medical conditions mimicking AAV, such as malignancies and significant infectious diseases requiring hospitalisation (3–5); 8) patients who had been exposed to immunosuppressive drugs for AAV treatment within 4 weeks before AAV diagnosis; and 9) patients who presented clinical symptoms suggestive of VPN and had the results of NCS performed within 3 months after AAV diagnosis (19–21). Of the 323 AAV patients, 204 were excluded from this study because they had no NCS results. Of the 119 AAV patients with available NCS results, 22 were excluded from this study because they underwent NCS after 3 months or more following AAV diagnosis. Finally, 97 patients presenting with clinical symptoms suggestive of VPN and with the results of NCS performed within 3 months after AAV diagnosis were included in this study (**Figure 1**). This study was approved by the Institutional Review Board (IRB) of Severance Hospital (Seoul, Korea, IRB No. 4-2020-1071) and conducted according to the Declaration of Helsinki. Due to the retrospective design of the study and the use of anonymised patient data, the requirement for written informed consent was waived.

### 2.2 Clinical data at AAV diagnosis

We collected variables recorded at the time of AAV as follows: 1) demographic data including age, sex, body mass index, and smoking history; 2) AAV-specific data including AAV subtype, ANCA type, indices for assessing activity and prognosis, and systemic involvement pattern; 3) laboratory data including erythrocyte sedimentation rate (ESR) and C-reactive protein (CRP); and 4) comorbidities affecting the results of NCS performed within 3 months after AAV diagnosis and poor outcomes during follow-up, such as type 2 diabetes mellitus, hypertension, and dyslipidaemia (23, 24). In terms of BVAS, because BVAS has an item containing a subitem of VPN, in cases where NCS was performed 1−3 months after diagnosis, the scores assigned to an item of nervous systemic manifestation were also added to the total BVAS at diagnosis retroactively (8). Conversely, because FFS has no neurological components, its retroactive application was unnecessary (9). MPO-ANCA and PR3-ANCA were measured using immunoassays, whereas P-ANCA and C-ANCA were evaluated using indirect immunofluorescence assays. The results of both assay methods were accepted as ANCA results according to the 2022 ACR/EULAR criteria for AAV (3–5).

### 2.3 Clinical features suggestive of VPN

In this study, the clinical symptoms suggestive of VPN included pain, tingling sensation, numbness, and/or weakness in the upper or lower extremities. VPN was classified by adopting the following criteria, where 1) AND 2) should be satisfied: 1) evidence of PN: i) electrodiagnostic evidence of an axonal neuropathy (on NCS) or ii) clinical neurologic examination findings of PN; AND 2) clinical presentation of PN: i) sensory and/or motor, plus ii) multifocal/asymmetric pattern without nerve root abnormalities, plus iii) either further clinical features including predominant symptoms in lower extremities, pain, and typical progress pattern, or biopsy findings (13). We categorised VPN into three groups according to the primary accompanying neurological types—sensory, motor, and mixed (sensory + motor)—and collected data regarding VPN types. Additionally, we reviewed the sites affected by VPN which were divided into seven main nerves: the median, ulnar, peroneal, posterior tibial, saphenous, sural, and plantar nerves. Because this was a retrospective study, NCS was performed as part of routine clinical practice rather than according to a prospectively standardized research protocol. NCS findings were reviewed from medical records. Clinical records were also reviewed to exclude evident alternative explanations for peripheral neuropathy, including nerve root compression, entrapment neuropathy, diabetic neuropathy, infection, malignancy, treatment-related neuropathy, or other mimicking conditions. The routine NCS protocol used at our institution has been described previously (25).

### 2.4 Histological description

Like the clinical information regarding VPN on NCS, data on the histological findings of nerve tissues were retrospectively collected from medical records. A critical clue for deciding the concordance or discordance of findings between NCS and nerve biopsy was based on the presence or absence of the terms ‘histological evidence of vasculitis’ after ‘axonopathy’.

### 2.5 Clinical data during follow-up

Medications administered to patients from the time of AAV diagnosis to the last visit were recorded. All-cause mortality (ACM), end-stage kidney disease (ESKD), cerebrovascular accident (CVA), and acute coronary syndrome (ACS) were investigated as poor AAV outcomes after diagnosis and during follow-up. For patients with each poor outcome, the follow-up duration based on each poor outcome was determined as the period from AAV diagnosis to each poor outcome occurrence. Meanwhile, for patients without each poor outcome, it was defined as the period from AAV diagnosis to the last visit.

### 2.6 Statistical analysis

All statistical analyses were performed using SPSS version 26 (IBM Corporation, Armonk, NY, USA) for Windows (Microsoft Corporation, Redmond, WA, USA). Continuous and categorical variables were expressed as a median (25–75 percentile), and a number (percentage). Significant differences between the two categorical variables were analysed using the chi-square and Fisher’s exact tests, whereas the continuous variables using the Mann-Whitney U test. A hazard ratio (HR) for each poor AAV outcome was obtained using Cox proportional hazard analyses. To address the potential confounding effect of diabetes mellitus on peripheral neuropathy and NCS findings, clinical and electrodiagnostic features were additionally compared between patients with and without type 2 diabetes mellitus. Statistical significance was set as P <0.05.

## 3 Results

### 3.1 Baseline characteristics

At the time of AAV diagnosis, patients were 63.0 (51.0─70.0) years old at median, and the percentage of men and women were 43% and 57%, respectively. Of the 97 patients, 48 patients were diagnosed with MPA, 20 patients with GPA, and 29 patients with EGPA. MPO-ANCA (or P-ANCA) and PR3-ANCA (or C-ANCA) were detected in 77 and 10 patients, respectively. The median BVAS and FFS were 12.0, and 1.0, respectively, and when investigated based on the items of BVAS, the most observed systemic manifestation was pulmonary (79.4%), followed by nervous systemic (67.0%), and renal (47.4%) manifestations. Among the laboratory results, the median ESR and CRP were 72.5 mm/h, and 16.2 mg/L, respectively. Other laboratory results and comorbidities are presented in **Table 1**.

**Table 1:** Baseline characteristics of AAV patients presenting clinical symptoms suggestive of VPN and having the results of NCS performed within 3 months after AAV diagnosis (N = 97).

Variables	AAV (N = 97)	MPA (N = 48)	GPA (N = 20)	EGPA (N = 29)	MPO-AAV (N = 75)	PR3-AAV (N = 8)
Demographic data						
Age (years)	63.0 (51.0─70.0)	67.5 (60.5–74.0)	58.0 (42.0–69.5)	52.0 (41.5–65.5)	66.0 (56.0–72.0)	52.5 (36.3–62.0)
Male sex [N, (%)]	42 (43.3)	22 (45.8)	8 (40.0)	12 (41.4)	33 (44.0)	3 (37.5)
Female sex [N, (%)]	55 (56.7)	26 (54.2)	12 (60.0)	17 (58.6)	42 (56.0)	5 (62.5)
BMI (kg/m^2^)	22.5 (20.2─24.2)	22.5 (20.8–23.7)	22.9 (20.4–25.7)	22.0 (19.4–25.3)	22.6 (20.8–24.1)	22.2 (20.1–25.4)
Ex-smoker [N, (%)]	4 (4.1)	1 (2.1)	1 (5.0)	2 (6.9)	3 (4.0)	0 (0.0)
AAV subtype [N, (%)]						
MPA	48 (49.5)	48 (100.0)	0 (0.0)	0 (0.0)	48 (64.0)	0 (0.0)
GPA	20 (20.6)	0 (0.0)	20 (100.0)	0 (0.0)	11 (14.7)	7 (87.5)
EGPA	29 (29.9)	0 (0.0)	0 (0.0)	29 (100.0)	16 (21.3)	1 (12.5)
ANCA type and positivity [N, (%)]						
MPO-ANCA (or P-ANCA) positivity	77 (79.4)	48 (100.0)	13 (65.0)	16 (55.2)	75 (100.0)	0 (0.0)
PR3-ANCA (or C-ANCA) positivity	10 (10.3)	0 (0.0)	9 (45.0)	1 (3.4)	0 (0.0)	8 (100.0)
Both ANCA positivity	2 (2.1)	0 (0.0)	2 (10.0)	0 (0.0)	0 (0.0)	0 (0.0)
ANCA negativity	12 (12.4)	0 (0.0)	0 (0.0)	12 (41.4)	0 (0.0)	0 (0.0)
AAV-specific indices						
BVAS	12.0 (7.5─19.0)	15.0 (8.3–19.0)	9.5 (7.3–15.0)	11.0 (7.0–18.5)	14.0 (8.0–19.0)	10.5 (7.3–19.3)
FFS	1.0 (0─2.0)	2.0 (1.0–2.0)	1.0 (0.0–2.0)	0.0 (0.0–1.0)	2.0 (1.0–2.0)	0.5 (0.0–1.0)
Systemic manifestations based on BVAS						
General	43 (44.3)	27 (56.3)	7 (35.0)	9 (31.0)	36 (48.0)	3 (37.5)
Cutaneous	14 (14.4)	6 (12.5)	1 (5.0)	7 (24.1)	10 (13.3)	1 (12.5)
Mucous and ocular	6 (6.2)	2 (4.2)	2 (10.0)	2 (6.9)	3 (4.0)	2 (25.0)
Otorhinolaryngological	44 (45.4)	10 (20.8)	10 (50.0)	24 (82.8)	26 (34.7)	6 (75.0)
Pulmonological	77 (79.4)	37 (77.1)	14 (70.0)	26 (89.7)	61 (81.3)	6 (75.0)
Cardiovascular	10 (10.3)	4 (8.3)	1 (5.0)	5 (17.2)	7 (9.3)	1 (12.5)
Gastrointestinal	3 (3.1)	1 (2.1)	0 (0.0)	2 (6.9)	2 (2.7)	0 (0.0)
Renal	46 (47.4)	35 (72.9)	8 (40.0)	3 (10.3)	43 (57.3)	2 (25.0)
Nervous systemic	65 (67.0)	29 (60.4)	15 (75.0)	21 (72.4)	50 (66.7)	5 (62.5)
Laboratory results						
White blood cell count (/mm^3^)	10,080.0 (7,095.0─13,720.0)	10,265.0 (6,497.5–15,097.5)	9,705.0 (6,927.5–12,857.5)	9,540.0 (7,280.0–13,775.0)	10,080.0 (6,830.0–13,640.0)	10,875.0 (8,315.0–13,637.5)
Haemoglobin (g/dL)	11.8 (9.9─13.3)	10.5 (9.0–12.3)	11.7 (9.8–12.5)	13.6 (12.4–14.6)	11.3 (9.5–12.8)	11.5 (9.5–13.1)
Platelet count (× 1000/mm^3^)	308.0 (232.0─396.5)	332.5 (236.0–392.0)	309.0 (230.8–431.3)	265.0 (211.0–405.0)	292.0 (228.5–383.0)	400.0 (284.0–454.3)
Blood urea nitrogen (mg/dL)	16.3 (12.4─24.7)	19.4 (14.9–33.4)	15.4 (11.8–26.3)	14.3 (9.6–18.1)	17.3 (13.3–27.8)	14.6 (11.6–25.1)
Serum creatinine (mg/dL)	0.7 (0.6─1.0)	0.8 (0.6–1.5)	0.7 (0.6–1.1)	0.7 (0.6–0.8)	0.7 (0.6–1.2)	0.7 (0.5–1.0)
Serum total protein (g/dL)	6.8 (6.1─7.3)	6.8 (6.1–7.2)	6.7 (5.9–7.3)	6.8 (6.2–7.5)	6.8 (6.1–7.2)	6.5 (6.0–7.2)
Serum albumin (g/dL)	3.7 (3.2─4.2)	3.3 (3.1–3.8)	3.8 (3.2–4.3)	3.8 (3.5–4.3)	3.6 (3.1–4.1)	3.7 (3.2–4.3)
ESR (mm/hr)	72.5 (26.0─118.8)	96.0 (55.0–120.0)	66.0 (24.0–95.0)	32.0 (11.0–74.3)	77.0 (36.5–120.0)	60.5 (12.5–100.3)
CRP (mg/L)	16.2 (1.9─76.5)	37.7 (3.5–117.6)	26.3 (1.0–65.3)	5.8 (0.9–18.0)	18.4 (1.9–79.2)	28.6 (6.6–79.1)
Albuminuria [N, (%)]		25 (52.1)	4 (20.0)	6 (20.7)	30 (40.0)	2 (25.0)
Haematuria [N, (%)]		18 (37.5)	4 (20.0)	6 (20.7)	24 (32.0)	3 (37.5)
Comorbidities [N, (%)]						
Type 2 diabetes mellitus	16 (16.5)	11 (22.9)	5 (25.0)	0 (0.0)	15 (20.0)	1 (12.5)
Hypertension	28 (28.9)	16 (33.3)	7 (35.0)	5 (17.2)	23 (30.7)	1 (12.5)
Dyslipidaemia	11 (11.3)	6 (12.5)	4 (20.0)	1 (3.4)	10 (13.3)	1 (12.5)

Values are expressed as a median (25–75 percentile) or N (%).

AAV, ANCA-associated vasculitis; ANCA, antineutrophil cytoplasmic antibody; NCS, nerve conduction studies; BMI, body mass index; MPA, microscopic polyangiitis; GPA, granulomatosis with polyangiitis; EGPA, eosinophilic granulomatosis with polyangiitis; MPO, myeloperoxidase; P, perinuclear; PR3, proteinase 3; C, cytoplasmic; BVAS, the Birmingham vasculitis activity score version 3; FFS, the five-factor score; ESR, erythrocyte sedimentation rate; CRP, C-reactive protein.

### 3.2 Proportion of NCS-classified VPN among symptomatic AAV patients who underwent early NCS

Among the 97 symptomatic AAV patients who underwent NCS within 3 months after AAV diagnosis, 55 patients (56.7%) were classified as having electrodiagnostically supported VPN according to the case definition and guidelines proposed in 2017 (13). All 97 patients had multifocal or asymmetric neuropathic symptoms, including pain, tingling sensation, numbness, and/or weakness in the upper or lower extremities, at any time during the first 3 months after AAV diagnosis. However, only 55 (56.7%) patients exhibited abnormal findings on NCS compatible with PN without evident alternative explanations such as infection, malignancy, or mechanical compression of nerve roots. The remaining 42 patients had neuropathic symptoms but did not show definite NCS evidence of peripheral neuropathy and were therefore not classified as having electrodiagnostically supported VPN. 12 of the 97 patients underwent nerve biopsy, and nine of them exhibited axonopathy with histological evidence of vasculitis (**Table 2**).

**Table 2:** Data regarding vasculitic peripheral neuropathy and NCS findings.

Variables						
.*Among patients with NCS results*	.AAV (N = 97)	.MPA (N = 48)	.GPA (N = 20)	.EGPA (N = 29)	.MPO-AAV (N = 75)	.PR3-AAV (N = 8)
Incidence of VPN (N, (%))	55 (56.7)	26 (54.2)	13 (65.0)	16 (55.2)	44 (58.7)	3 (37.5)
Clinical features suggestive of VPN [N, (%)] (N = 97)						
I. Evidence of PN						
a. electrodiagnostic evidence of an axonal neuropathy	55 (56.7)	26 (54.2)	13 (65.0)	16 (55.2)	44 (58.7)	3 (37.5)
OR						
b. clinical examination signs of PN^*^	N/A	N/A	N/A	N/A	N/A	N/A
AND						
II. Clinical presentation typical for VPN						
a. Sensory-motor or sensory (not pure motor)^**^	97 (100)	48 (100)	20 (100)	29 (100)	75 (100)	8 (100)
AND						
b. Multifocal or asymmetric pattern at any time, AND this is not attributable to compression or entrapment of peripheral nerves or roots	97 (100)	48 (100)	20 (100)	29 (100)	75 (100)	8 (100)
AND						
c. EITHER						
i. Further clinical features:						
(1) Lower limb predominant	97 (100)	48 (100)	20 (100)	29 (100)	75 (100)	8 (100)
AND						
(2) Painful	97 (100)	48 (100)	20 (100)	29 (100)	75 (100)	8 (100)
AND						
(3) One or more acute attacks, or variable speed of progression, or improvement of motor of sensory deficit	97 (100)	48 (100)	20 (100)	29 (100)	75 (100)	8 (100)
OR						
ii. Biopsy or nerve shows histopathologically probable vasculitis (N = 12)	9 of 12 (75.0)	5 of 12 (41.7)	2 of 12 (16.7)	5 of 12 (41.7)	7 of 12 (58.3)	1 of 12 (8.3)
.*Among patients with VPN on NCS*	.AAV (N = 55)	.MPA (N = 48)	.GPA (N = 20)	.EGPA (N = 29)	.MPO-AAV (N = 75)	.PR3-AAV (N = 8)
NCS findings						
Types of VPN (N, (%))						
Mixed	46 (83.6)	22 (45.8)	9 (45.0)	15 (51.7)	37 (49.3)	3 (37.5)
Pure sensory	9 (16.4)	4 (8.3)	4 (20.0)	1 (3.4)	7 (9.3)	0 (0.0)
Pure motor	0 (0)	0 (0)	0 (0)	0 (0)	0 (0)	0 (0)
Sites affected by VPN (N, (%))						
Median nerve	26 (47.3)	13 (27.1)	3 (15.0)	10 (34.5)	22 (29.3)	0 (0.0)
Ulnar nerve	26 (47.3)	16 (33.3)	3 (15.0)	7 (24.1)	24 (32.0)	0 (0.0)
Peroneal nerve	39 (70.9)	21 (43.8)	9 (45.0)	9 (31.0)	31 (41.3)	3 (37.5)
Posterior tibial nerve	36 (65.5)	19 (39.6)	5 (25.0)	12 (41.4)	30 (40.0)	2 (25.0)
Saphenous nerve	3 (5.5)	2 (4.2)	1 (5.0)	0 (0.0)	2 (2.7)	0 (0.0)
Sural nerve	28 (50.9)	16 (33.3)	5 (25.0)	7 (24.1)	25 (33.3)	2 (25.0)
Plantar nerve	3 (5.5)	1 (2.1)	1 (5.0)	1 (3.4)	1 (1.3)	1 (12.5)

Values are expressed as N (%).

* Because of the retrospective study design, neurological examination findings corresponding to this item could not be collected from all patients.

** Sensory neuropathic symptoms were found in all 97 patients who underwent NCS regardless of the presence of motor neuropathic manifestation.

NCS, nerve conduction studies; VPN, vasculitic peripheral neuropathy; PN, peripheral neuropathy; AAV, ANCA-associated vasculitis; ANCA, antineutrophil cytoplasmic antibody; N/A, not applicable.

### 3.3 Clinical characteristics of VPN in AAV patients with NCS results

The clinical characteristics of the patients revealed that the most common VPN type was the mixed type, and the most frequently affected site was the peroneal nerve. Among the 55 patients with AAV with NCS findings suggesting VPN, 46 (83.6%) had the mixed type of VPN, and the remaining nine had the pure sensory type of VPN. The peroneal nerve was the most frequently affected (70.9%), followed by the posterior tibial nerve (65.5%) (**Table 2**).

### 3.4 Comparison of baseline variables between patients with VPN on NCS and those without

No significant differences were observed in baseline demographic data, AAV subtypes, ANCA positivity, laboratory results, or comorbidities between the two groups. Conversely, patients with VPN on NCS showed significantly higher initial BVAS and FFS than those without (15.0 vs. 10.0, P = 0.010, and 2.0 vs. 1.0, P = 0.007). A higher frequency of nervous systemic manifestation in patients with VPN on NCS compared to those without was natural. However, a relatively lower incidence of mucous/ocular manifestation in those with VPN than those without was noticeable (1.8% vs. 11.9%, P = 0.082) (**Table 3**). Detailed comparisons of baseline variables between patients with and without VPN on NCS in each AAV subtype and MPO-AAV are shown in **Supplementary Tables 1** and **2**, respectively.

**Table 3:** Comparison of baseline variables of AAV patients having the results of NCS performed within 3 months after AAV diagnosis according to VPN (N = 97).

Variables	AAV patients without VPN on NCS (N = 42)	AAV patients with VPN on NCS (N = 55)	P-value
Demographic data			
Age (years)	59.5 (53.8─69.0)	66.0 (50.0─72.0)	0.484
Male sex [N, (%)]	21 (50.0)	21 (38.2)	0.244
Female sex [N, (%)]	21 (50.0)	34 (61.8)	
BMI (kg/m^2^)	22.5 (20.9─25.3)	22.4 (20.1─23.7)	0.333
Ex-smoker [N, (%)]	1 (2.4)	3 (5.5)	0.631
AAV subtype [N, (%)]			0.700
MPA	22 (52.4)	26 (47.3)	
GPA	7 (16.7)	13 (23.6)	
EGPA	13 (31.0)	16 (29.1)	
ANCA type and positivity [N, (%)]			
MPO-ANCA (or P-ANCA) positivity	31 (73.8)	46 (83.6)	0.236
PR3-ANCA (or C-ANCA) positivity	5 (11.9)	5 (9.1)	0.652
Both ANCA positivity	0 (0)	2 (3.6)	0.504
ANCA negativity	6 (14.3)	6 (10.9)	0.617
AAV-specific indices			
BVAS	10.0 (6.8─16.3)	15.0 (9.0─21.0)	0.010
FFS	1.0 (0─2.0)	2.0 (1.0─2.0)	0.007
Systemic manifestations based on BVAS			
General	18 (42.9)	25 (45.5)	0.799
Cutaneous	7 (16.7)	7 (12.7)	0.584
Mucous and ocular	5 (11.9)	1 (1.8)	0.082
Otorhinolaryngological	23 (54.8)	21 (38.2)	0.104
Pulmonological	36 (85.7)	41 (74.5)	0.178
Cardiovascular	3 (7.1)	7 (12.7)	0.507
Gastrointestinal	0 (0)	3 (5.5)	0.256
Renal	18 (42.9)	28 (50.9)	0.431
Nervous systemic	10 (23.8)	55 (100)	<0.001
Laboratory results			
White blood cell count (/mm^3^)	9,995.0 (7,000.0─12,407.5)	10,280.0 (6,970.0─14,930.0)	0.256
Haemoglobin (g/dL)	12.1 (9.5─13.5)	11.3 (9.9─13.1)	0.572
Platelet count (× 1000/mm^3^)	343.5 (226.5─403.0)	306.0 (235.0─395.0)	0.455
Blood urea nitrogen (mg/dL)	15.9 (12.1─23.1)	16.7 (12.5─26.0)	0.867
Serum creatinine (mg/dL)	0.8 (0.6─1.2)	0.7 (0.6─1.0)	0.158
Serum total protein (g/dL)	6.8 (6.4─7.4)	6.8 (6.0─7.2)	0.320
Serum albumin (g/dL)	3.7 (3.3─4.1)	3.6 (3.1─4.2)	0.373
ESR (mm/hr)	71.0 (32.0─112.0)	75.0 (19.0─120.0)	0.870
CRP (mg/L)	14.1 (3.8─63.5)	19.2 (1.0─78.3)	0.535
Comorbidities [N, (%)]			
Type 2 diabetes mellitus	5 (11.9)	11 (20.0)	0.287
Hypertension	14 (33.3)	14 (25.5)	0.396
Dyslipidaemia	4 (9.5)	7 (12.7)	0.752

Values are expressed as a median (25–75 percentile) or N (%).

AAV, ANCA-associated vasculitis; ANCA, antineutrophil cytoplasmic antibody; NCS, nerve conduction studies; VPN, vasculitic peripheral neuropathy; BMI, body mass index; MPA, microscopic polyangiitis; GPA, granulomatosis with polyangiitis; EGPA, eosinophilic granulomatosis with polyangiitis; MPO, myeloperoxidase; P, perinuclear; PR3, proteinase 3; C, cytoplasmic; BVAS, the Birmingham vasculitis activity score version 3; FFS, the five-factor score; ESR, erythrocyte sedimentation rate; CRP, C-reactive protein.

To address the potential confounding effect of type 2 diabetes mellitus on NCS findings, we additionally compared clinical and electrodiagnostic features according to diabetes mellitus status. Patients with type 2 diabetes mellitus were older and had higher BVAS, more frequent renal involvement, lower haemoglobin levels, and higher ESR and CRP levels than those without type 2 diabetes mellitus. However, the proportion of NCS-classified VPN was not significantly different between patients with and without type 2 diabetes mellitus (68.8% vs. 54.3%, P = 0.409), and the distributions of major electrodiagnostic findings were also comparable between the two groups (**Supplementary Table 3**).

### 3.5 Discordance of findings between NCS and nerve biopsy

NCS and nerve biopsy provided complementary information in the evaluation of possible VPN. The concordance rate of findings suggestive of VPN between the two groups was 75.0%. Among the 12 patients who underwent nerve biopsy, three exhibited discordances between the two findings because no histological evidence of vasculitis was found despite NCS findings suggestive of VPN. All three patients showing the discrepancy were classified as having EGPA (three of the five EGPA patients) (**Table 4**). Of the three patients, one patient was MPO-ANCA positive, whereas the other two patients were ANCA negative.

**Table 4:** Concordance or discordance of findings between NCS and nerve biopsy.

Patient number N/97 (N/323)	AAV subtype	ANCA positivity	Vasculitic peripheral neuropathy on NCV	Vasculitis on histology	Concordance or discordance	Biopsy site	Histological findings
2 (11)	MPA	MPO-ANCA	Yes	Yes	Concordance	Left sural nerve	Axonopathy with vasculitis
5 (34)	MPA	MPO-ANCA	Yes	Yes	Concordance	Left sural nerve	Axonopathy with vasculitis
13 (109)	GPA	PR3-ANCA	Yes	Yes	Concordance	Left peroneal nerve	Axonopathy with vasculitis Perineural fibrosis
21 (166)	EGPA	Negative	Yes	Yes	Concordance	Right sural nerve	Axonopathy with vasculitis Eosinophilic degranulation
31 (203)	EGPA	Negative	Yes	No	Discordance	Right sural nerve	Axonopathy without histological evidence of vasculitis Need for clinical correlation
34 (210)	MPA	Negative	Yes	Yes	Concordance	Left dorsal ulnar cutaneous nerve	Axonopathy with vasculitis
47 (232)	GPA	MPO-ANCA	Yes	Yes	Concordance	Right sural nerve	Axonopathy with vasculitis and vessel injury and damage
56 (247)	EGPA	MPO-ANCA	Yes	No	Discordance	Left sural nerve	Axonopathy without histological evidence of vasculitis Need for clinical correlation
59 (252)	MPA	MPO-ANCA	Yes	Yes	Concordance	Left sural nerve	Axonopathy with vasculitis
77 (288)	EGPA	Negative	Yes	No	Discordance	Left dorsal ulnar cutaneous nerve	Axonopathy without histological evidence of vasculitis Need for clinical correlation
81 (292)	EGPA	MPO-ANCA	Yes	Yes	Concordance	Left sural nerve	Axonopathy with vasculitis with eosinophil infiltration
89 (307)	MPA	MPO-ANCA	Yes	Yes	Concordance	Left sural nerve	Axonopathy with vasculitis (partial healed vasculitis)

NCS, nerve conduction studies; AAV, ANCA-associated vasculitis; ANCA, antineutrophil cytoplasmic antibody; MPA, microscopic polyangiitis; GPA, granulomatosis with polyangiitis; EGPA, eosinophilic granulomatosis with polyangiitis.

### 3.6 Comparison of baseline variables between patients with peripheral neuropathy on NCS according to histological evidence of vasculitis

Patients with histological evidence of vasculitis exhibited a higher female sex ratio, whereas a lower frequency of otorhinolaryngological manifestation (all P = 0.045) than those without. Particularly, patients with vasculitis confirmed on nerve biopsy had a higher median ESR than those without (81.0 vs. 6.0 mm/h, P = 0.009) (**Table 5**).

**Table 5:** Comparison of baseline variables between patients with histological evidence of vasculitis and those without (N = 12).

Variables	AAV patients with VPN on NCS but without histological evidence of vasculitis (N = 3)	AAV patients with VPN on NCS and with histological evidence of vasculitis (N = 9)	P-value
Demographic data			
Age (years)	55.0 (47.0─73.0)	63.0 (38.5─67.5)	0.864
Male sex [N, (%)]	2 (66.7)	0 (0)	0.045
Female sex [N, (%)]	1 (33.3)	9 (100)	
BMI (kg/m^2^)	23.4 (21.3─24.2)	23.2 (22.3─25.2)	1.000
Ex-smoker [N, (%)]	1 (33.3)	0 (0)	0.250
AAV subtype [N, (%)]			0.061
MPA	0 (0)	5 (55.6)	
GPA	0 (0)	2 (22.2)	
EGPA	3 (100)	2 (22.2)*	
ANCA type and positivity [N, (%)]			
MPO-ANCA (or P-ANCA) positivity	1 (33.3)	7 (77.8)	0.236
PR3-ANCA (or C-ANCA) positivity	0 (0)	1 (11.1)	1.000
Both ANCA positivity	0 (0)	0 (0)	N/A
ANCA negativity	2 (66.7)	1 (11.1)	0.127
AAV-specific indices			
BVAS	9.0 (8.0─25.0)	12.0 (8.5─19.0)	1.000
FFS	0 (0─1.0)	1.0 (0.5─2.5)	0.145
Systemic manifestations based on BVAS			
General	1 (33.3)	5 (55.6)	1.000
Cutaneous	0 (0)	2 (22.2)	1.000
Mucous and ocular	0 (0)	0 (0)	N/A
Otorhinolaryngological	3 (100)	2 (22.2)	0.045
Pulmonological	2 (66.7)	2 (22.2)	0.236
Cardiovascular	1 (33.3)	0 (0)	0.250
Gastrointestinal	0 (0)	1 (11.1)	1.000
Renal	0 (0)	2 (22.2)	1.000
Nervous systemic	3 (100)	9 (100)	N/A
Laboratory results			
White blood cell count (/mm^3^)	7,340.0 (6,400.0─9,320.0)	10,950.0 (6,865.0─19,365.0)	0.282
Haemoglobin (g/dL)	14.5 (10.3─14.6)	12.3 (10.1─13.4)	0.282
Platelet count (× 1000/mm^3^)	172.0 (171.0─459.0)	331.0 (299.0─379.0)	0.373
Blood urea nitrogen (mg/dL)	18.1 (10.1─18.3)	16.5 (15.0─21.3)	1.000
Serum creatinine (mg/dL)	0.5 (0.5─0.9)	0.5 (0.5─0.7)	0.600
Serum total protein (g/dL)	6.0 (4.7─7.0)	6.6 (6.3─7.4)	0.209
Serum albumin (g/dL)	4.0 (2.6─4.4)	3.5 (3.1─4.0)	0.864
ESR (mm/hr)	6.0 (3.0─11.0)	81.0 (30.5─111.5)	0.009
CRP (mg/L)	1.1 (0.5─11.9)	30.8 (1.0─98.3)	0.282
Comorbidities [N, (%)]			
Type 2 diabetes mellitus	0 (0)	2 (22.2)	1.000
Hypertension	0 (0)	2 (22.2)	1.000
Dyslipidaemia	0 (0)	1 (11.1)	1.000

Values are expressed as a median (25–75 percentile) or N (%).

*Both EGPA patients in this group were ANCA negative.

AAV, ANCA-associated vasculitis; ANCA, antineutrophil cytoplasmic antibody; NCS, nerve conduction studies; BMI, body mass index; MPA, microscopic polyangiitis; GPA, granulomatosis with polyangiitis; EGPA, eosinophilic granulomatosis with polyangiitis; MPO, myeloperoxidase; P, perinuclear; PR3, proteinase 3; C, cytoplasmic; BVAS, the Birmingham vasculitis activity score version 3; FFS, the five-factor score; ESR, erythrocyte sedimentation rate; CRP, C-reactive protein.

### 3.7 Medications and poor outcomes

Regarding the medications administered, no significant differences were found between the two groups according to VPN in the results of NCS. The incidence rates of poor AAV outcomes including ACM, ESKD, CVA, and ACS did not differ between the two groups (**Supplementary Table 4**). Additionally, in univariable Cox analysis, baseline VPN identified on NCS was not significantly associated with any poor outcome during follow-up in AAV patients (**Supplementary Table 5**).

## 4 Discussion

In this study, we included only patients presenting with clinical symptoms suggestive of VPN and with available results of NCS performed within 3 months after AAV diagnosis. We described the proportion and clinical characteristics of electrodiagnostically supported VPN in this selected symptomatic cohort. We obtained several findings in this study. First, 5 of 97 patients (56.7%) were classified as having NCS-supported VPN. Second, the most common type of VPN was the mixed type (67.3%), and the most frequently affected nerve was the peroneal nerve (70.9%). Third, patients with VPN on NCS had significantly higher initial BVAS and FFS than those without. The higher BVAS appeared to be primarily attributable to the nervous system component of the BVAS, as no significant differences were observed in the involvement of the other organ systems. Fourth, among the 12 AAV patients who underwent nerve biopsy, the concordance rate between NCS and nerve biopsy findings was 75% based on histological evidence of vasculitis. Finally, among the 12 AAV patients who underwent nerve biopsy, nine patients with histological evidence of vasculitis showed a higher ESR level and a lower frequency of ear, nose, and throat symptoms than the three patients without. Therefore, we believe that this study suggests a potential adjunctive role of NCS in identifying VPN and supporting differential diagnosis in AAV patients complaining of clinical symptoms of VPN in the extremities. NCS and nerve biopsy provide complementary information in the evaluation of possible VPN. NCS helps identify objective peripheral nerve involvement and characterize the electrodiagnostic pattern, whereas nerve biopsy provides histological confirmation of vasculitic involvement when clinically necessary and feasible. Therefore, NCS may be used as a non-invasive diagnostic tool in symptomatic patients, while nerve biopsy should be considered selectively when histological confirmation is required or when the diagnosis remains uncertain.

In the present study, we pragmatically determined the cut-off interval between AAV diagnosis and NCS performance at 3 months and included only patients with the available results of NCS performed within 3 months after AAV diagnosis in the analyses. Accordingly, 22 patients were excluded from the analysis despite having NCS results. These inclusion/exclusion criteria were selected to capture early peripheral nerve involvement around the time of AAV diagnosis while reducing, as much as possible, the potential influence of induction treatment administered after diagnosis on NCS findings. Although this 3-month interval has not been externally validated as a definitive cut-off for evaluating VPN in AAV, it was considered clinically reasonable based on the following considerations. First, in representative previous clinical studies, the periods during which significant therapeutic effects after the initiation of induction therapy could be confirmed were approximately 2−3 months (26, 27). Second, based on the dosing schedule of rituximab or cyclophosphamide as a monotherapy for remission-induction, and the pharmacological mechanism of each drug, meaningful and effective immune cell depletion and/or modulation have been observed approximately three months after the initiation of induction therapy (26, 28). Third, in a previous study, the period during which vasculitis activity decreased to <50% after induction therapy in AAV patients exhibiting PN was approximately 3 months (29). Therefore, we selected the 3-month interval as a pragmatic early assessment window, while acknowledging that treatment effects and disease evolution during this period could not be completely excluded.

Regarding the sensory and motor nerves affected by AAV, in this study, the most common type of VPN was the mixed type (83.6%), followed by the pure sensory type. In actual clinical practice, there may be AAV patients with the pure motor type, but this type was not found in any of the 55 patients. As pure motor involvement is not accepted by these criteria, this was an expected result based on the proposed clinical presentation of VPNs (13). Regarding the site of nerves affected by AAV, the peroneal, tibial, and sural nerves showed higher frequencies of VPN than the median or ulnar nerves. Whereas, from a clinical point of view of VPN prognosis and convenience of evaluation, when categorised into upper and lower extremities rather than by specific nerves affected by VPN, this study revealed that the lower extremities showed a higher VPN frequency than the upper extremities. Based on these results, we suggest that NCS be performed in AAV patients who complain of sensory abnormalities in the lower extremities.

In the comparative analysis according to VPN identified on NCS, patients with VPN exhibited higher BVAS and FFS than those without. Because BVAS included an item of nervous systemic manifestation, it was naturally accepted that BVAS was significantly higher in patients with VPN as much as the score assigned to PN (8). In addition, analysis of the individual BVAS organ manifestations demonstrated that, apart from the expected difference in nervous system involvement, no distinct organ involvement clustering pattern was identified. However, we did not anticipate that FFS, which consists of age ≥65 years, cardiac insufficiency, renal insufficiency, gastrointestinal involvement, and absence of ear, nose, and throat manifestations, was higher in patients with VPN. When the frequency of the five components of FFS was indirectly compared through the results in **Table 3**, patients with VPN showed a tendency toward otorhinolaryngological manifestations at a lower frequency than patients without (38.2% vs. 54.8%, P = 0.104) (9). Using a receiver operating characteristic curve, the cut-off of FFS for the presence of VPN was extrapolated as 2, which had the maximum sum of sensitivity and specificity. When AAV patients were divided into two groups according to FFS of 2, VPN was found more frequently in patients with FFS ≥2 than those with FFS <2 (P = 0.027). Additionally, patients with FFS ≥2 exhibited a higher relative risk (RR) for VPN compared to those with FFS <2 (RR 2.593, 95% confidence interval 1.105, 6.084) (**Supplementary Figure 1**). Although the cut-off value of FFS for VPN cannot be generalized to AAV patients due to the small number of patients included in this study, this result may help determine whether NCS should performed considering NCS in patients with ambiguous symptoms suspected of VPN.

Additionally, among the nine systemic manifestations based on BVAS except for nervous systemic manifestations, mucous/ocular manifestations were found in patients with VPN identified on NCS relatively commonly compared to those without VPN (P = 0.082). Given that the frequencies of both mucous/ocular and otorhinolaryngological manifestations in patients with VPN increased, it could be reasonably presumed that VPN occurring in AAV patients might be associated with symptoms corresponding to GPA surrogate markers for the upper respiratory tract, as suggested by the EMA algorithm for AAV, despite no difference in the frequency of GPA between the two groups (2). However, the exact mechanism underlying this assumption could not be proposed.

Among the 97 patients with NCS results, only 12 with VPN confirmed on NCS underwent nerve biopsy. Of these, nine showed axonopathy with vasculitis and the remaining three exhibited axonopathy without histological evidence of vasculitis, resulting in a discordance rate of 25.0%. Notably, all three discordant cases occurred in patients with EGPA, including one MPO-ANCA-positive patient and two ANCA-negative patients. However, because of the very small number of discordant cases, the clinical significance of ANCA status should be interpreted with caution. Although the findings of cross-sectional vasculitis in the nerve tissues of the three patients were not prominent, it was reasonable to recognise them as patients with VPN for the following reasons. First, the observation of axonopathy suggests that it was preceded by ischaemic injury due to vasculitis or a vasculitis-related inflammatory process (30, 31). Second, owing to the limitations of the nerve tissue obtained through biopsy, revealing all existing vasculitis was not possible. Therefore, even if the histological evidence of vasculitis is not definite, it can be sufficiently assumed to be VPN because it meets the criteria for VPN (13). Third, considering that ESR was significantly higher in patients with confirmed vasculitis as shown in **Table 5**, the presence or absence of vasculitis may be transient, and the concept of old and current vasculitis should also be considered. Although axonopathy alone was insufficient to establish histologically supported VPN in the absence of vascular or perivascular inflammation, NCS findings compatible with peripheral neuropathy in a clinically suggestive context supported the classification as having electrodiagnostically supported VPN, highlighting the complementary roles of NCS and nerve biopsy.

The strength of this study is that it investigated the proportion and clinical characteristics of VPN by selectively including only patients with NCS results performed in the earlier stage of AAV when the effects of therapeutic drugs on the classification of VPN were negligible. Furthermore, this study suggested the potential adjunctive role of NCS in VPN classification in patients who underwent both NCS and nerve biopsy despite the small number of patients.

### 4.1 Limitations

This study has several limitations. First, the relatively small number of patients in this single-centre cohort, particularly the small number of patients who underwent NCS, and the retrospective study design were the primary limitations. Second, as we analysed only 97 patients who underwent NCS among the 323 patients with AAV, we could not determine the overall frequency of VPN in all AAV patients. Third, the 3-month interval between AAV diagnosis and NCS performance was selected as a pragmatic cut-off to capture early peripheral nerve involvement while reducing the potential influence of later treatment exposure; however, this cut-off has not been externally validated, and the effects of treatment and disease evolution during this period cannot be completely excluded. Forth, the low proportion of patients who underwent nerve biopsy may have limited the reliability of the analysis of the concordance between NCS and nerve biopsy findings. Concomitant muscle biopsy was not performed, which may have limited the diagnostic yield of histological evaluation for vasculitic peripheral neuropathy. Fifth, AAV is a heterogeneous disease spectrum, and neurological manifestations may differ among MPA, GPA, and EGPA and according to ANCA specificity. Because formal subgroup analyses were not performed owing to the limited sample size of some subgroups, the present findings should be interpreted as results from a heterogeneous AAV cohort rather than as subtype-specific estimates. Sixth, vasculitic activity at diagnosis was assessed using BVAS. However, persistent vasculitic activity after diagnosis was not systematically analysed as a separate early outcome parameter. Seventh, although we additionally compared clinical and electrodiagnostic features according to type 2 diabetes mellitus status, residual confounding by diabetes mellitus, disease severity, treatment exposure, and other comorbidities cannot be excluded. Finally, NCS was not performed serially, before and after treatment or during the worsening and improvement of symptoms. A future prospective and multi-centre study with more patients and serial NCS assessments is expected to provide more reliable and valuable information on the clinical utility of NCS for the classification of VPN in AAV patients.

### 4.2 Conclusion

In this selected cohort of symptomatic AAV patients who underwent NCS within 3 months after diagnosis, more than half were classified as having electrodiagnostically supported VPN. NCS may provide adjunctive objective information for characterizing peripheral nerve involvement in clinically suspected VPN.

## Data Availability

The raw data supporting the conclusions of this article will be made available by the authors, without undue reservation.

## Supplementary material

Supplementary Figure 1 ROC curve analysis of FFS and its relative risk for VPN in patients with AAV. **(A)** In ROC curve analysis, FFS exhibited a significant area under the curve for VPN. **(B)** Patients with FFS ≥2 exhibited a higher relative risk for VPN than those without. ROC, receive operating characteristic; FFS, the five-factor score; VPN, vasculitic peripheral neuropathy; AAV, antineutrophil cytoplasmic antibody-associated vasculitis.
