# Supplementary material for: Clinical and electrodiagnostic features of nerve conduction study-classified peripheral neuropathy in symptomatic patients with antineutrophil cytoplasmic antibody-associated vasculitis: a single-centre cohort study
Source: Front Immunol. 2026 Jul 30;17:1807774. doi: 10.3389/fimmu.2026.1807774 (PMC13468926; doi:10.3389/fimmu.2026.1807774)
Supplement: Supplementary file 1 [file Table1.docx]

**Supplementary Table 1. Comparison of baseline variables between those without and with VPN on NCS in each AAV subtype**

| **Variables** | **MPA patients without**  **VPN on NCS (N=22)** | **MPA patients with**  **VPN on NCS (N=26)** | **P-value** | **GPA patients without**  **VPN on NCS (N=7)** | **GPA patients with**  **VPN on NCS (N=13)** | **P-value** | **EGPA patients without**  **VPN on NCS (N=13)** | **EGPA patients with**  **VPN on NCS (N=16)** | **P-value** |
| --- | --- | --- | --- | --- | --- | --- | --- | --- | --- |
| **Demographic data** |  |  |  |  |  |  |  |  |  |
| Age (years) | 66.0 (59.8–72.5) | 68.0 (61.3–74.0) | 0.656 | 40.0 (35.0–57.0) | 66.0 (54.0–72.5) | 0.002 | 55.0 (51.5–66.0) | 48.5 (38.5–64.0) | 0.121 |
| Male sex (N, (%)) | 11 (50.0) | 11 (42.3) | 0.594 | 3 (42.9) | 5 (38.5) | >0.999 | 7 (53.8) | 5 (31.3) | 0.219 |
| Female sex (N, (%)) | 11 (50.0) | 15 (57.7) | 0.594 | 4 (57.1) | 8 (61.5) | >0.999 | 6 (46.2) | 11 (68.8) | 0.219 |
| BMI (kg/m^2^) | 22.7 (21.7–24.5) | 21.5 (20.1–23.3) | 0.172 | 21.7 (20.1–27.4) | 23.0 (21.2–24.1) | 0.817 | 22.2 (19.4–27.1) | 21.7 (19.1–24.7) | 0.503 |
| Ex-smoker (N, (%)) | 1 (4.5) | 0 (0.0) | 0.458 | 0 (0.0) | 1 (7.7) | >0.999 | 0 (0.0) | 2 (12.5) | 0.488 |
| **AAV subtype (N, (%))** |  |  |  |  |  |  |  |  |  |
| MPA | 22 (100.0) | 26 (100.0) | N/A | 0 (0.0) | 0 (0.0) | N/A | 0 (0.0) | 0 (0.0) | N/A |
| GPA | 0 (0.0) | 0 (0.0) | N/A | 7 (100.0) | 13 (100.0) | N/A | 0 (0.0) | 0 (0.0) | N/A |
| EGPA | 0 (0.0) | 0 (0.0) | N/A | 0 (0.0) | 0 (0.0) | N/A | 13 (100.0) | 16 (100.0) | N/A |
| **ANCA type and positivity (N, (%))** |  |  |  |  |  |  |  |  |  |
| MPO-ANCA (or P-ANCA) positivity | 22 (100.0) | 26 (100.0) | N/A | 3 (42.9) | 10 (76.9) | 0.174 | 6 (46.2) | 10 (62.5) | 0.379 |
| PR3-ANCA (or C-ANCA) positivity | 0 (0.0) | 0 (0.0) | N/A | 4 (57.1) | 5 (38.5) | 0.642 | 1 (7.7) | 0 (0.0) | 0.448 |
| Both ANCA positivity | 0 (0.0) | 0 (0.0) | N/A | 0 (0.0) | 2 (15.4) | 0.521 | 0 (0.0) | 0 (0.0) | N/A |
| ANCA negativity | 0 (0.0) | 0 (0.0) | N/A | 0 (0.0) | 0 (0.0) | N/A | 6 (16.2) | 6 (37.5) | 0.638 |
| **AAV-specific indices** |  |  |  |  |  |  |  |  |  |
| BVAS | 14.5 (5.8–18.0) | 16.5 (10.5–22.3) | 0.075 | 9.0 (8.0–14.0) | 10.0 (7.0–17.0) | 0.699 | 9.0 (6.5–13.5) | 14.5 (8.3–23.3) | 0.050 |
| FFS | 1.5 (0.8–2.0) | 2.0 (1.8–3.0) | 0.016 | 0.0 (0.0–1.0) | 1.0 (1.0–2.0) | 0.097 | 0.0 (0.0–1.0) | 0.5 (0.0–1.0) | 0.268 |
| **Systemic manifestations based on BVAS** |  |  |  |  |  |  |  |  |  |
| General | 15 (68.2) | 12 (46.2) | 0.125 | 0 (0.0) | 7 (53.8) | 0.044 | 3 (23.1) | 6 (37.5) | 0.454 |
| Cutaneous | 2 (9.1) | 4 (15.4) | 0.674 | 0 (0.0) | 1 (7.7) | >0.999 | 5 (38.5) | 2 (12.5) | 0.192 |
| Mucous and ocular | 2 (9.1) | 0 (0.0) | 0.205 | 2 (28.6) | 0 (0.0) | 0.111 | 1 (7.7) | 1 (6.3) | >0.999 |
| Otorhinolaryngological | 7 (31.8) | 3 (11.5) | 0.152 | 4 (57.1) | 6 (46.2) | >0.999 | 12 (92.3) | 12 (75.0) | 0.343 |
| Pulmonological | 18 (81.8) | 19 (73.1) | 0.473 | 6 (85.7) | 8 (61.5) | 0.354 | 12 (92.3) | 14 (87.5) | >0.999 |
| Cardiovascular | 1 (4.5) | 3 (11.5) | 0.614 | 1 (14.3) | 0 (0.0) | 0.350 | 1 (7.7) | 4 (25.0) | 0.343 |
| Gastrointestinal | 0 (0.0) | 1 (3.8) | >0.999 | 0 (0.0) | 0 (0.0) | N/A | 0 (0.0) | 2 (12.5) | 0.488 |
| Renal | 16 (72.7) | 19 (73.1) | 0.978 | 2 (28.6) | 6 (46.2) | 0.642 | 0 (0.0) | 3 (18.8) | 0.232 |
| Nervous systemic | 3 (13.6) | 26 (100.0) | <0.001 | 2 (28.6) | 13 (100.0) | 0.001 | 5 (38.5) | 16 (100.0) | <0.001 |
| **Laboratory results** |  |  |  |  |  |  |  |  |  |
| White blood cell count (/mm^3^) | 10,075.0 (5,622.5–13,095.0) | 10,980.0 (7,240.0–15,507.5) | 0.185 | 10,500.0 (8,640.0–12,400.0) | 7,500.0 (5,815.0–13,220.0) | 0.393 | 8,850.0 (6,780.0–11,955.0) | 10,020.0 (7,602.5–19,495.0) | 0.249 |
| Haemoglobin (g/dL) | 10.1 (8.7–12.7) | 10.7 (9.8–11.8) | 0.514 | 11.9 (11.0–12.6) | 11.3 (9.0–12.7) | 0.757 | 14.2 (13.4–14.7) | 13.3 (12.1–14.5) | 0.232 |
| Platelet count (× 1000/mm^3^) | 345.0 (219.8–395.0) | 324.0 (238.0–390.5) | 0.772 | 373.0 (364.0–458.0) | 292.0 (202.5–365.5) | 0.037 | 253.0 (195.0–378.5) | 286.5 (251.3–408.5) | 0.531 |
| Blood urea nitrogen (mg/dL) | 19.8 (15.5–37.7) | 17.8 (12.7–29.3) | 0.612 | 11.9 (11.5–22.8) | 15.6 (13.0–28.6) | 0.351 | 13.7 (9.5–16.5) | 14.6 (9.3–19.6) | 0.531 |
| Serum creatinine (mg/dL) | 0.9 (0.7–2.0) | 0.8 (0.6–1.4) | 0.296 | 0.7 (0.6–1.2) | 0.7 (0.5–1.6) | 0.817 | 0.8 (0.6–0.9) | 0.7 (0.5–0.8) | 0.170 |
| Serum total protein (g/dL) | 6.7 (6.2–7.2) | 6.8 (6.0–7.1) | 0.686 | 7.0 (6.5–7.4) | 6.4 (5.6–7.3) | 0.393 | 6.8 (6.5–7.5) | 6.9 (6.0–7.5) | 0.682 |
| Serum albumin (g/dL) | 3.5 (3.1–4.0) | 3.3 (3.1–3.8) | 0.582 | 3.9 (3.2–4.4) | 3.7 (3.1–4.3) | 0.817 | 4.1 (3.7–4.5) | 3.8 (3.2–4.3) | 0.249 |
| ESR (mm/hr) | 91.0 (50.5–120.0) | 103.5 (64.8–120.0) | 0.601 | 89.0 (37.0–95.0) | 39.5 (18.8–95.3) | 0.311 | 32.0 (9.8–62.8) | 36.5 (11.0–80.0) | 0.589 |
| CRP (mg/L) | 25.6 (8.7–111.5) | 63.0 (1.9–118.1) | 0.548 | 26.3 (13.2–50.4) | 24.6 (0.6–68.5) | 0.773 | 3.4 (1.2–10.2) | 8.8 (0.6–22.0) | 0.664 |
| **Comorbidities (N, (%))** |  |  |  |  |  |  |  |  |  |
| Type 2 diabetes mellitus | 4 (18.2) | 7 (26.9) | 0.473 | 1 (14.3) | 4 (30.8) | 0.613 | 0 (0.0) | 0 (0.0) | N/A |
| Hypertension | 9 (40.9) | 7 (26.9) | 0.306 | 1 (14.3) | 6 (46.2) | 0.329 | 4 (30.8) | 1 (6.3) | 0.144 |
| Dyslipidaemia | 3 (13.6) | 3 (11.5) | >0.999 | 1 (14.3) | 3 (23.1) | >0.999 | 0 (0.0) | 1 (6.3) | >0.999 |

Values are expressed as a median (25-75 percentile) or N (%).

AAV: ANCA-associated vasculitis; ANCA: antineutrophil cytoplasmic antibody; NCS: nerve conduction studies; VPN: vasculitic peripheral neuropathy; BMI: body mass index; MPA: microscopic polyangiitis; GPA: granulomatosis with polyangiitis; EGPA: eosinophilic granulomatosis with polyangiitis; MPO: myeloperoxidase; P: perinuclear; PR3: proteinase 3; C: cytoplasmic; BVAS: the Birmingham vasculitis activity score version 3; FFS: the five-factor score; ESR: erythrocyte sedimentation rate; CRP: C-reactive protein.
