# Supplementary material for: Clinical and electrodiagnostic features of nerve conduction study-classified peripheral neuropathy in symptomatic patients with antineutrophil cytoplasmic antibody-associated vasculitis: a single-centre cohort study
Source: Front Immunol. 2026 Jul 30;17:1807774. doi: 10.3389/fimmu.2026.1807774 (PMC13468926; doi:10.3389/fimmu.2026.1807774)
Supplement: Supplementary file 2 [file Table2.docx]

**Supplementary Table 2. Comparison of baseline variables between those without and with VPN on NCS in MPO-AAV and PR3-AAV patients**

| **Variables** | **MPO-AAV patients without**  **VPN on NCS (N=31)** | **MPO-AAV patients with**  **VPN on NCS (N=44)** | **P-value** |
| --- | --- | --- | --- |
| **Demographic data** |  |  |  |
| Age (years) | 63.0 (56.0–69.0) | 67.5 (51.3–73.8) | 0.471 |
| Male sex (N, (%)) | 16 (51.6) | 17 (38.6) | 0.265 |
| Female sex (N, (%)) | 15 (48.4) | 27 (61.4) | 0.265 |
| BMI (kg/m^2^) | 22.7 (21.8–24.9) | 22.2 (19.9–23.7) | 0.234 |
| Ex-smoker (N, (%)) | 1 (3.2) | 2 (4.5) | >0.999 |
| **AAV subtype (N, (%))** |  |  |  |
| MPA | 22 (71.0) | 26 (59.1) | 0.498 |
| GPA | 3 (9.7) | 8 (18.2) | 0.498 |
| EGPA | 6 (19.4) | 10 (22.7) | 0.498 |
| **ANCA type and positivity (N, (%))** |  |  |  |
| MPO-ANCA (or P-ANCA) positivity | 31 (100.0) | 44 (100.0) | N/A |
| PR3-ANCA (or C-ANCA) positivity | 0 (0.0) | 0 (0.0) | N/A |
| Both ANCA positivity | 0 (0.0) | 0 (0.0) | N/A |
| ANCA negativity | 0 (0.0) | 0 (0.0) | N/A |
| **AAV-specific indices** |  |  |  |
| BVAS | 11.0 (6.0–16.0) | 15.0 (9.0–19.8) | 0.024 |
| FFS | 1.0 (0.0–2.0) | 2.0 (1.0–2.8) | 0.014 |
| **Systemic manifestations based on BVAS** |  |  |  |
| General | 17 (54.8) | 19 (43.2) | 0.320 |
| Cutaneous | 3 (9.7) | 7 (15.9) | 0.509 |
| Mucous and ocular | 2 (6.5) | 1 (2.3) | 0.566 |
| Otorhinolaryngological | 14 (45.2) | 12 (27.3) | 0.109 |
| Pulmonological | 25 (80.6) | 36 (81.8) | 0.898 |
| Cardiovascular | 1 (3.2) | 6 (13.6) | 0.228 |
| Gastrointestinal | 0 (0.0) | 2 (4.5) | 0.508 |
| Renal | 18 (58.1) | 25 (56.8) | 0.914 |
| Nervous systemic | 6 (19.4) | 44 (100.0) | <0.001 |
| **Laboratory results** |  |  |  |
| White blood cell count (/mm^3^) | 10,070.0 (6,130.0–12,430.0) | 10,245.0 (7,032.5–14,675.0) | 0.312 |
| Haemoglobin (g/dL) | 12.0 (8.9–12.9) | 11.3 (9.7–12.5) | 0.698 |
| Platelet count (× 1000/mm^3^) | 325.0 (225.0–387.0) | 290.0 (230.5–365.0) | 0.494 |
| Blood urea nitrogen (mg/dL) | 17.3 (12.5–29.9) | 17.1 (13.6–29.6) | 0.983 |
| Serum creatinine (mg/dL) | 0.8 (0.6–1.7) | 0.7 (0.6–1.0) | 0.180 |
| Serum total protein (g/dL) | 6.7 (6.4–7.3) | 6.8 (5.9–7.2) | 0.431 |
| Serum albumin (g/dL) | 3.7 (3.3–4.1) | 3.5 (3.1–4.1) | 0.457 |
| ESR (mm/hr) | 74.0 (39.5–120.0) | 83.5 (34.3–120.0) | 0.536 |
| CRP (mg/L) | 16.2 (3.8–59.0) | 21.0 (1.0–92.1) | 0.552 |
| **Comorbidities (N, (%))** |  |  |  |
| Type 2 diabetes mellitus | 5 (16.1) | 10 (22.7) | 0.482 |
| Hypertension | 11 (35.5) | 12 (27.3) | 0.448 |
| Dyslipidaemia | 3 (9.7) | 7 (15.9) | 0.509 |

Values are expressed as a median (25-75 percentile) or N (%).

AAV: ANCA-associated vasculitis; ANCA: antineutrophil cytoplasmic antibody; NCS: nerve conduction studies; VPN: vasculitic peripheral neuropathy; BMI: body mass index; MPA: microscopic polyangiitis; GPA: granulomatosis with polyangiitis; EGPA: eosinophilic granulomatosis with polyangiitis; MPO: myeloperoxidase; P: perinuclear; PR3: proteinase 3; C: cytoplasmic; BVAS: the Birmingham vasculitis activity score version 3; FFS: the five-factor score; ESR: erythrocyte sedimentation rate; CRP: C-reactive protein.
