# Supplementary material for: Clinical and electrodiagnostic features of nerve conduction study-classified peripheral neuropathy in symptomatic patients with antineutrophil cytoplasmic antibody-associated vasculitis: a single-centre cohort study
Source: Front Immunol. 2026 Jul 30;17:1807774. doi: 10.3389/fimmu.2026.1807774 (PMC13468926; doi:10.3389/fimmu.2026.1807774)
Supplement: Supplementary file 3 [file Table3.docx]

**Supplementary Table 3. Comparison of clinical and electrodiagnostic features according to type 2 diabetes mellitus status in symptomatic AAV patients who underwent early NCS**

| **Variables** | **AAV patients without**  **type 2 diabetes mellitus (N=81)** | **AAV patients with**  **type 2 diabetes mellitus (N=16)** | **P-value** |
| --- | --- | --- | --- |
| **Demographic data** |  |  |  |
| Age (years) | 59.0 (50.8-69.0)) | 68.0 (67.0-73.0) | <0.001 |
| Male sex (N, (%)) | 35 (43.2) | 7 (43.8) | 0.968 |
| Female sex (N, (%)) | 46 (56.8) | 9 (56.3) |  |
| BMI (kg/m^2^) | 22.7 (20.8-24.3) | 22.2 (19.6-23.4) | 0.230 |
| Ex-smoker (N, (%)) | 3 (3.7) | 1 (6.3) | 0.520 |
| **AAV subtype (N, (%))** |  |  | 0.007 |
| MPA | 37 (45.7) | 11 (68.8) |  |
| GPA | 15 (18.5) | 5 (31.3) |  |
| EGPA | 29 (35.8) | 0 (0) |  |
| **ANCA type and positivity (N, (%))** |  |  |  |
| MPO-ANCA (or P-ANCA) positivity | 62 (76.5) | 15 (93.8) | 0.120 |
| PR3-ANCA (or C-ANCA) positivity | 9 (11.1) | 1 (6.3) | >0.999 |
| Both ANCA positivity | 2 (2.5) | 0 (0) | >0.999 |
| ANCA negativity | 12 (14.8) | 0 (0) | 0.207 |
| **AAV-specific indices** |  |  |  |
| BVAS | 12.0 (7.0-18.0) | 15.0 (9.0-23.0) | 0.031 |
| FFS | 1.0 (0-2.0) | 2.0 (1.0-3.0) | 0.073 |
| **Systemic manifestations based on BVAS** |  |  |  |
| General | 35 (43.2) | 8 (50.0) | 0.617 |
| Cutaneous | 12 (14.8) | 2 (12.5) | >0.999 |
| Mucous and ocular | 5 (6.2) | 1 (6.3) | >0.999 |
| Otorhinolaryngological | 36 (44.4) | 8 (50.0) | 0.683 |
| Pulmonological | 64 (79.0) | 13 (81.3) | >0.999 |
| Cardiovascular | 10 (12.3) | 0 (0) | 0.206 |
| Gastrointestinal | 2 (2.5) | 1 (6.3) | 0.421 |
| Renal | 34 (42.0) | 12 (75.0) | 0.026 |
| Nervous systemic | 53 (65.4) | 12 (75.0) | 0.568 |
| **Laboratory results** |  |  |  |
| White blood cell count (/mm^3^) | 10,230.0 (6,935.0-13,810.0) | 9,510.0 (6,080.0-12,400.0) | 0.443 |
| Haemoglobin (g/dL) | 12.1 (10.2-13.4) | 10.0 (8.6-12.8) | 0.017 |
| Platelet count (× 1000/mm^3^) | 300.0 (233.5-388.5) | 366.0 (227.0-420.0) | 0.206 |
| Blood urea nitrogen (mg/dL) | 16.5 (12.5-26.2) | 16.3 (11.8-24.2) | 0.723 |
| Serum creatinine (mg/dL) | 0.7 (0.6-1.0) | 0.7 (0.6-1.2) | 0.926 |
| Serum total protein (g/dL) | 6.7 (6.1-7.3) | 7.0 (6.6-7.3) | 0.257 |
| Serum albumin (g/dL) | 3.7 (3.2-4.1) | 3.4 (3.2-4.0) | 0.579 |
| ESR (mm/hr) | 66.0 (25.5-103.3) | 104.0 (75.0-120.0) | 0.017 |
| CRP (mg/L) | 10.7 (1.8-55.9) | 65.7 (14.1-104.3) | 0.042 |
| **Comorbidities (N, (%))** |  |  |  |
| Hypertension | 20 (24.7) | 8 (50.0) | 0.041 |
| Dyslipidaemia | 7 (8.6) | 4 (25.0) | 0.080 |
| **NCS findings** |  |  |  |
| **NCS-classified VPN (N, (%))** | 44 (54.3) | 11 (68.8) | 0.409 |
| **Types of VPN (N, (%))** |  |  |  |
| Mixed | 37 (45.7) | 9 (56.3) | 0.439 |
| Pure sensory | 7 (8.6) | 2 (12.5) | 0.640 |
| Pure motor | 0 (0) | 0 (0) |  |
| **Sites affected by VPN (N, (%))** |  |  |  |
| Median nerve | 20 (24.7) | 6 (37.5) | 0.356 |
| Ulnar nerve | 21 (25.9) | 5 (31.3) | 0.759 |
| Peroneal nerve | 31 (38.3) | 8 (50.0) | 0.382 |
| Posterior tibial nerve | 29 (35.8) | 7 (43.8) | 0.548 |
| Saphenous nerve | 2 (2.5) | 1 (6.3) | 0.421 |
| Sural nerve | 22 (27.2) | 6 (37.5) | 0.546 |
| Plantar nerve | 1 (1.2) | 2 (12.5) | 0.070 |

Values are expressed as a median (25-75 percentile) or N (%).

AAV: ANCA-associated vasculitis; ANCA: antineutrophil cytoplasmic antibody; NCS: nerve conduction studies; VPN: vasculitic peripheral neuropathy; BMI: body mass index; MPA: microscopic polyangiitis; GPA: granulomatosis with polyangiitis; EGPA: eosinophilic granulomatosis with polyangiitis; MPO: myeloperoxidase; P: perinuclear; PR3: proteinase 3; C: cytoplasmic; BVAS: the Birmingham vasculitis activity score version 3; FFS: the five-factor score; ESR: erythrocyte sedimentation rate; CRP: C-reactive protein.
