# Supplementary material for: Clinical and electrodiagnostic features of nerve conduction study-classified peripheral neuropathy in symptomatic patients with antineutrophil cytoplasmic antibody-associated vasculitis: a single-centre cohort study
Source: Front Immunol. 2026 Jul 30;17:1807774. doi: 10.3389/fimmu.2026.1807774 (PMC13468926; doi:10.3389/fimmu.2026.1807774)
Supplement: Supplementary file 4 [file Table4.docx]

**Supplementary Table 4.** **Comparison of medications administered and AAV poor outcomes during follow-up according to VPN on NCS**

| **Variables** | **Values**  **(N=97)** | **AAV patients without**  **VPN on NCS (N=42)** | **AAV patients with**  **VPN on NCS (N=55)** | **P-value** |
| --- | --- | --- | --- | --- |
| **Medications (N, (%))** |  |  |  |  |
| **Induction** |  |  |  |  |
| Glucocorticoids | 94 (96.9) | 39 (92.9) | 55 (100) | 0.078 |
| Rituximab* | 61 (62.9) | 13 (31.0) | 11 (20.0) | 0.215 |
| Cyclophosphamide* | 24 (24.7) | 24 (57.1) | 37 (67.3) | 0.306 |
| Plasma exchange | 13 (13.4) | 6 (14.3) | 7 (12.7) | 0.823 |
| **Maintenance** |  |  |  |  |
| Mycophenolate mofetil | 29 (29.9) | 13 (31.0) | 16 (29.1) | 0.843 |
| Azathioprine | 59 (60.8) | 24 (57.1) | 35 (63.6) | 0.516 |
| Tacrolimus | 8 (8.2) | 4 (9.5) | 4 (7.3) | 0.724 |
| Methotrexate | 14 (14.4) | 6 (14.3) | 8 (14.5) | 0.971 |
| **Poor outcomes (N, (%))** |  |  |  |  |
| ACM | 12 (12.4) | 4 (9.5) | 8 (14.5) | 0.545 |
| ESKD | 6 (6.2) | 2 (4.8) | 4 (7.3) | 0.695 |
| CVA | 7 (7.2) | 2 (4.8) | 5 (9.1) | 0.695 |
| ACS | 1 (1.0) | 0 (0) | 1 (1.8) | 1.000 |

Values are expressed as a median (25-75 percentile) or N (%).

*Rituximab or cyclophosphamide was used as monotherapy for induction remission, and not in combination.

AAV: ANCA-associated vasculitis; ANCA: antineutrophil cytoplasmic antibody; VPN: vasculitic peripheral neuropathy; NCS: nerve conduction studies; ACM: all-cause mortality; ESKD: end-stage kidney disease; CVA: cerebrovascular accident; ACS: acute coronary syndrome.
