# Supplementary material for: Clinical and electrodiagnostic features of nerve conduction study-classified peripheral neuropathy in symptomatic patients with antineutrophil cytoplasmic antibody-associated vasculitis: a single-centre cohort study
Source: Front Immunol. 2026 Jul 30;17:1807774. doi: 10.3389/fimmu.2026.1807774 (PMC13468926; doi:10.3389/fimmu.2026.1807774)
Supplement: Supplementary file 5 [file Table5.docx]

**Supplementary Table 5. Univariable Cox proportional hazards analysis of VPN on NCS performed within 3 months after AAV diagnosis (N=97)**

| **Variables** | **HR** | **95% CI** | **P-value** |
| --- | --- | --- | --- |
| ACS | 1.494 | 0.449, 4.969 | 0.512 |
| ESKD | 1.598 | 0.293, 8.724 | 0.589 |
| CVA | 1.968 | 0.382, 10.146 | 0.418 |
| ACS | 50.795 | 0.000, 617,976,365.330 | 0.637 |

VPN: vasculitic peripheral neuropathy; NCS: nerve conduction studies; AAV: ANCA-associated vasculitis; ANCA: antineutrophil cytoplasmic antibody; ACM: all-cause mortality; ESKD: end-stage kidney disease; CVA: cerebrovascular accident; ACS: acute coronary syndrome.
